# Personals

**Published:** 1914-01

**Authors:** 


### PERSONALS

---

*Announcement of removal, travel, and other matters of interest are requested. Please report errors in the listing of any physician in the State and other directories that we may co-operate with the State Society in securing a correct list.*

---

Dr. Roswell Park of Buffalo addressed the University Club of Buffalo, Dec. 6, 1913, on The Purpose and Work of the New State Hospital for the Study of Malignant Disease, giving also a talk on the general nature of cancer, which he considers as probably due to microorganism.

---

Dr. Stephen Yates Howell of Buffalo has returned from a trip around the world lasting ten months.

Dr. L. M. Wilkins of Lackawanna was quite seriously injured, Nov. 27, by a collision between his automobile and a street car.

---

Miss Jessie Broadhurst, late of the Hospital of the Good Shepherd of Syracuse, has been appointed superintendent of the Broad Street Hospital of Oneida.

---

Dr. Julius H. Potter of Buffalo took a trip to St. Louis in December.

---

Dr. John A. Ragone of Buffalo sailed Dec. 11 for Europe, expecting to spend six months in various medical centers.

---

Dr. Clayton M. Brown of Buffalo has returned from Europe.

---

Dr. Mary I. Denton of the Western College, Oxford, O., a former Buffalonian, visited in Buffalo in December.

---

Dr. Hal B. Brownell of Buffalo has returned from a trip to Georgia.

---

Dr. Wm. L. Phillips of Buffalo has returned from a four months' European trip.

---

Dr. John D. O'Brien of Buffalo spent Thanksgiving in Ashtabula.

---

Dr. F. M. Boyle of Buffalo has been offered the position of First Deputy to the newly appointed Commissioner of Public Charities for Erie County, Mr. Wm. Hunt. The salary is $3,000.

---

Dr. H. H. Glosser has moved to 404 Franklin street, Buffalo, a few doors south of his former residence.

---

Dr. H. C. Leonhardt, formerly of North Tonawanda, who has spent the last nine years in Florida, is located at 267 Franklin street, Buffalo.

